# Federal Food Assistance Program Participation during the COVID-19 Pandemic: Participant Perspectives and Reasons for Discontinuing

**DOI:** 10.3390/nu14214524

**Published:** 2022-10-27

**Authors:** Emily M. Melnick, Montserrat Ganderats-Fuentes, Punam Ohri-Vachaspati

**Affiliations:** College of Health Solutions, Arizona State University, Phoenix, AZ 85004, USA

**Keywords:** SNAP, WIC, COVID-19

## Abstract

This study aims to describe reasons for discontinuing participation and experiences participating in the Supplemental Nutrition Assistance Program (SNAP) and the Special Supplemental Nutrition Assistance Program for Women, Infants, and Children (WIC) during the COVID-19 pandemic. We analyzed data from a cross-sectional online survey distributed to a national sample, restricted to (1) households that discontinued participating in SNAP (n = 146) or WIC (n = 149) during the pandemic and (2) households that participated in SNAP (n = 501) or WIC (n = 141) during spring 2021—approximately one year into the pandemic. We conducted thematic analyses of open-ended survey questions and descriptive statistics for Likert-scale items. Themes raised by respondents who discontinued participating in SNAP or WIC included difficulty recertifying and virus exposure concerns. Former WIC participants reported the program was not worth the effort and former SNAP participants reported failing to requalify. Respondents participating in WIC or SNAP during the pandemic mentioned transportation barriers and insufficient benefit value. WIC participants had trouble redeeming benefits in stores and SNAP participants desired improved online grocery purchasing experiences. These results suggest that enhancements to WIC and SNAP, such as expanded online purchasing options, program flexibilities, and benefit increases, can improve program participation to ensure access to critical nutrition supports, especially during emergencies.

## 1. Introduction

Following the onset of the global COVID-19 pandemic in 2020, rates of unemployment, poverty, and food insecurity rose dramatically [1,2]. Accompanying mitigation efforts to curb COVID-19 transmission have also affected food chains, resulting in food shortages and rising food prices [3]. Federal nutrition assistance programs provide vital nutrition support in this economic climate for millions of Americans by supplementing their food budget.

The largest federal nutrition assistance program in the US is the Supplemental Nutrition Assistance Program (SNAP). Households qualify for SNAP based on financial need and receive an Electronic Benefit Transfer (EBT) card that can be used to purchase groceries from authorized stores. In the fiscal year 2020, 39.8 million income-eligible Americans received SNAP benefits each month, receiving an average monetary benefit value of USD 154.81 per person [4]. The Special Supplemental Nutrition Program for Women, Infants, and Children (WIC) is another federal program that provides support for pregnant women and children under the age of 5 in the form of monthly supplemental nutritious foods that can be purchased from authorized stores. In 2020, 6.2 million individuals participated in WIC, receiving an average monthly benefit of USD 38.48 per person [5].

Despite the health and economic benefits federal nutrition assistance programs provide, accessibility barriers, compounded by the challenges presented by the COVID-19 pandemic, limited individuals’ use of these safety nets. Understanding barriers to program participation during this public health emergency is critical to informing future program and policy adaptations that can help improve participation and retention within these assistance programs, especially during periods when communities are faced with disaster situations. To examine participant experiences during the pandemic, we sought to answer two questions: (1) why did some individuals who received SNAP or WIC benefits the year prior to the COVID-19 pandemic stop participating? and (2) what were the experiences of those individuals participating in SNAP or WIC programs approximately 1 year into the COVID-19 pandemic? Our analytic approach, which combines a qualitative analysis of open-ended questions and a quantitative analysis of closed-ended questions, provides in-depth information on food assistance program participation experiences of households at-risk of food and nutrition insecurity during the COVID-19 pandemic.

## 2. Materials and Methods

### 2.1. Survey Instrument

An online survey was administered electronically to a national sample in the spring of 2021, including an oversampling of lower-income individuals (total n = 1967). The survey used a combination of closed-ended and open-ended questions asking respondents about food security, food access, and food assistance program participation during COVID-19. The survey was conducted as a part of the National Food Access and COVID Research Team (NFACT) study, which brought together researchers from multiple states in the US to conduct research on food access during the COVID-19 pandemic. The survey instrument used for the current study is publicly available [6]. A full description of the NFACT study is available elsewhere [7].

Relevant to the current analysis, the survey assessed SNAP and WIC program participation. Respondents were asked, “Which of the following food assistance programs did your household use in the last 4 months/in the year before the COVID-19 outbreak?”. Response options included a list of food assistance programs with an option to check boxes for all programs that applied. The analytic sample for research question #1 (why did some individuals who received SNAP or WIC benefits the year prior to the COVID-19 pandemic stop participating?) includes individuals who reported that they participated in SNAP or WIC the year prior to the onset of the COVID-19 pandemic and reported that they did not participate in the last 4 months (spanning between November 2020 and May 2021 based on the date of survey completion). Based on branching survey logic, if respondents reported that they participated in a program in the year prior to COVID-19 and did not participate in the same program in the last 4 months, these respondents (SNAP n = 146, WIC n = 149) were asked to “Tell us more about why you did not participate in SNAP/WIC in the last 4 months.”

The analytic sample for research question #2 (what were the experiences of those individuals participating in SNAP or WIC programs approximately 1 year into the COVID-19 pandemic?) included individuals who reported participating in SNAP (n = 501) or in WIC (n = 141) in the 4 months prior to the survey, regardless of participation prior to the onset of the pandemic. Based on branching survey logic, respondents who reported participating in these programs were asked 5 Likert-scale questions to assess their experience using these programs over the prior 4 months. In addition to Likert-scale questions, all respondents who reported participating in SNAP or WIC were asked the open-ended question: “Any other comments about using SNAP/WIC during the COVID-19 outbreak?” Additional open-ended questions were displayed to respondents based on responses to closed-ended items. If respondents answered “Agree” or “Strongly agree” to “We are not able to use our full months’ worth of SNAP/WIC benefits”, they were asked to “Tell us more about why you are unable to use your full months’ worth of SNAP/WIC benefits.” If respondents answered “Agree” or “Strongly agree” to “We cannot use SNAP benefits to pay for groceries ordered online”, to follow up, they were asked to “Tell us more about your experience not being able to use SNAP benefits online”.

### 2.2. Data Collection

The online survey was open between 24 March 2021 and 6 May 2021. Survey participants were recruited from Qualtrics (Provo, UT) online panels and included individuals ≥ 18 years of age living in the US to reflect the US adult population by race/ethnicity and age based on the 2018 American Community Survey data (5-year estimates). Individuals from lower-income households (i.e., those having a household income below USD 50,000 in 2019) were oversampled. The survey was available in English and Spanish, and the average completion time was 23 min. Respondents viewed an informed consent form containing information about the study, the voluntary nature of their participation, how their results would be kept confidential, and contact information for a study team member in the case that they had any questions concerning the study before answering survey questions. Respondents indicated consent to participate by clicking “yes” to begin taking the survey. As part of the informed consent, respondents were informed that if they completed the survey, they would receive points directly from their Qualtrics panel provider and, in addition, would be entered into a raffle with a 1 in 20 chance of winning a USD 25 gift card to a grocery store. The study was approved by the authors’ Institutional Review Board through an expedited review procedure.

### 2.3. Data Analysis

Open-ended questions were analyzed using an iterative thematic approach by 2 researchers with prior qualitative data analysis experience. As a first step, the researchers independently read and re-read open-ended survey questions. Next, researchers independently developed an initial set of codes to describe respondent perceptions based on commonly occurring themes they noticed. The researchers subsequently met and developed a code book including names and definitions for agreed upon final codes. Researchers then independently assigned codes to all open-ended responses using the code book. Intercoder agreement at this stage was high; disagreement occurred on 13.1% of total codes. If discordance on codes occurred, a discussion occurred to reach consensus on the final codes assigned to each quote. Descriptive statistics were calculated for Likert-scale items displayed to respondents who participated in SNAP/WIC during the 4 months prior the study using Stata Software (Statacorp LLC, version 16.1).

## 3. Results

The final sample consisted of 803 unique respondents living within 47 different states in the US. Households can participate in SNAP and WIC concurrently; therefore, respondents could offer responses about both programs, individually. Among the subsample of respondents who discontinued participating in SNAP (n = 146) or WIC (n = 149) during COVID-19 after participating in the year prior to the onset of the outbreak, 58 reported having discontinued participation in both programs. Of these, 19 respondents provided separate open-ended responses describing reasons for discontinuing participation in each program. Among the subsample of respondents currently participating in SNAP (n = 504) or WIC (n = 141), 76 reported currently participating in both programs. Of these, four respondents provided separate open-ended responses describing their experiences participating in each program.

Race was self-reported by respondents from a list including White, Black or African American, American Indian or Alaskan Native, Asian Indian, Chinese, Filipino, Japanese, Korean, Native Hawaiian, Samoan, Vietnamese, or other (specify). Respondents also self-reported whether they were “of Hispanic, Latino, or Spanish origin” (yes/no). Table 1 presents self-reported demographic data for: (1) respondents who participated in SNAP or WIC prior to the onset of the COVID-19 pandemic and did not participate during the pandemic in the 4 months prior to survey completion, and (2) respondents who participated in SNAP and WIC during the pandemic in the 4 months prior to survey completion, hereafter referred to as current participants.

Among respondents who discontinued participating in SNAP, 18.5% identified as Hispanic and 11.6% identified as non-Hispanic Black. Race/ethnicity distribution was similar among current participants. A majority of current and former SNAP participants reported having a household income of less than USD 50,000, though a greater percentage of current SNAP participants reported incomes below USD 50,000 compared to those who discontinued participating (86.6% vs. 61.3%). Among respondents who discontinued participating in WIC, 24.8% identified as Hispanic and 14.8% identified as non-Hispanic Black. Among current WIC participants, 17.7% identified as Hispanic and 10.6% identified as non-Hispanic Black. A majority of current and former WIC participants reported having a household income of less than USD 50,000 (64.7% of current participants and 69.0% of those who discontinued participating). 

Through open-ended and closed-ended survey items, participants shared their (A) reasons for discontinuing SNAP or WIC participation during COVID-19 pandemic and (B) experiences participating in SNAP or WIC during COVID-19. 

### 3.1. A. Reasons for Discontinuing SNAP or WIC Participation during COVID-19 Pandemic 

Thematic analyses yielded overarching themes capturing individuals’ reasons for discontinuing participating in SNAP or WIC during the COVID-19 pandemic. These themes emerging from open-ended responses are summarized in Table 2 and are designated in both the table and in text as either “shared” (reported in relation to both SNAP and WIC programs) or “unique” (distinctive to either SNAP or WIC):

- **Shared Theme A.1. Virus exposure concerns.** A recurring worry described by individuals who discontinued participation in SNAP or WIC was a fear of exposure to the COVID-19 virus. Respondents reported fears of virus exposure while redeeming food benefits in stores; for example, a former SNAP participant wrote, “Not COVID safe to go in store”. Some respondents who discontinued participating in WIC shared that they chose to discontinue participating because they did not want to visit program clinics for in-person appointments: “*Did not want to risk going in a small room with lots of others.*”
- **Shared Theme A.2. Difficulty with recertification processes**. Respondents who formerly participated in SNAP or WIC described barriers to recertifying eligibility following the conclusion of their active certified period. Two subthemes about recertification processes emerged: time and technology. Respondents from both programs shared that they discontinued participating because they did not have the time to fill out paperwork to recertify eligibility. A respondent who previously participated in WIC wrote “*I didn’t want to have to requalify and didn’t have the time.”* In addition, experiencing technical challenges and being *“unable to get signed up online”* was shared as a barrier.
- **Shared Theme A.3. Saving for others.** A final shared theme was a desire to save benefits “for the less fortunate families” who respondents perceived needed it more. As an example, a former SNAP participant wrote, *“Because I and my family [sic] has enough these few months and we felt it wasn’t right to shortchange people”*.
- **Unique SNAP Theme A.4. Fail to requalify.** In addition to other expressed barriers, some respondents reported that they were deemed ineligible to continue receiving SNAP benefits, despite believing they should still qualify for assistance. A respondent shared, *“I’m disabled but my income went down due to Medicare kicking in, but I went from being qualified for the max to not qualifying as I made to [sic] much money and got kicked off SNAP... I don’t know how my income went down but I went from getting max benefit to making too much”.*
- **Unique WIC Theme A.5. Perception that program is not worth the effort.** A theme unique to former WIC participants was a perception that the program was no longer needed or that the effort required to participate was not sustainable. For example, a mother reported discontinuing participation in WIC because, *“my kids no longer need formula, so I dropped it”*. Another expressed that it was *“just too complicated”* to remain enrolled in WIC.

### 3.2. B. What Were the Experiences of Individuals Participating in SNAP or WIC Programs during the COVID-19 Pandemic?

Themes and subthemes emerging from thematic analysis of open-ended items exploring the experiences of individuals participating in SNAP or WIC during the COVID-19 pandemic are summarized in Table 2 and described in text.

- **Shared Theme B.1. Insufficient benefit value.** Current participants in both SNAP and WIC shared that the benefit value they received was not enough to meet their households’ monthly needs. For example, a SNAP participant shared that they *“Wish there was more money”*, and a WIC participant wrote *“the little they give, it’s not enough”*. Some SNAP respondents raised specific concerns about how benefit amounts and emergency allotments were calculated during the pandemic. A SNAP participant wrote, “they should have extended the SNAP benefits for everyone”.
- **Shared Theme B.2. Transportation barriers.** Another shared barrier was difficulty traveling to an eligible retailer to purchase groceries using benefits. Respondents noted challenges including a lack of gas money, a vehicle, and a lack of time to travel to stores and shop for groceries. A SNAP participant wrote, *“I don’t have a vehicle right now, so I can only get to a grocery store if I pay for a rideshare service. The rates for that ride are so high that I just can’t afford that. If I were able to pay for groceries online with my EBT card and get grocery delivery, I wouldn’t have to worry about that”*. Some WIC participants mentioned how few stores near their homes were approved retailers, increasing the difficulty of redeeming their benefits.
- **Unique SNAP Theme B.3. Difficulty with online grocery purchasing**. Many SNAP participants described trouble finding retailers that accepted payment using Electronic Benefits Transfer (EBT) cards for online grocery shopping. For example, a respondent shared *“No stores except [sic] snap for online groceries. It’s very inconvenient when your [sic] disabled and stuck at home in quarantine”*. Two additional subthemes emerged within this overarching theme. First, the inability to buy online food increased participant fears of COVID-19 virus exposure; a respondent wrote *“it was difficult because my family is high risk for COVID-19 and we found it difficult to order contactless pick up/delivery groceries due to them being unavailable to SNAP users”*. Second, participants described difficulty acquiring fresh foods online using SNAP benefits.
- **Unique SNAP Theme B.4. Increased benefits are useful**. A number of SNAP participants described the USDA emergency allotments they received during the pandemic [8] as helpful. One participant wrote *“the extra amount they have given during the pandemic has really helped alot [sic] & we greatly appreciate it.”* Another shared *“the additional EBT money given during the pandemic is the only thing that stopped me from going hungry”*.
- **Unique WIC Theme B.5. Difficulty finding eligible items in store**. Many WIC participants expressed that they encountered challenges finding WIC-eligible foods in stock in stores, in large part due to food supply chain disruptions occurring during the pandemic. One WIC participant communicated this struggle, writing *“during the pandemic there wasn’t enough food stocked”*. Relatedly, WIC participants expressed a desire for *“a wider variety of foods and brands”* as allowable foods they could purchase with their benefits because it was “hard to find the WIC items” during the pandemic.

**Figure 1:**
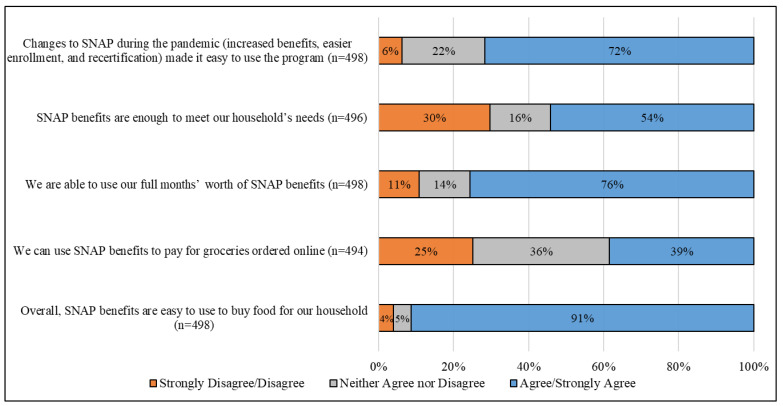
Perceptions of the Supplemental Nutrition Assistance Program (SNAP) use during the Coronavirus Disease 2019 (COVID-19) pandemic ^†^. ^†^ Respondent numbers across questions vary due to non-response.

**Figure 2:**
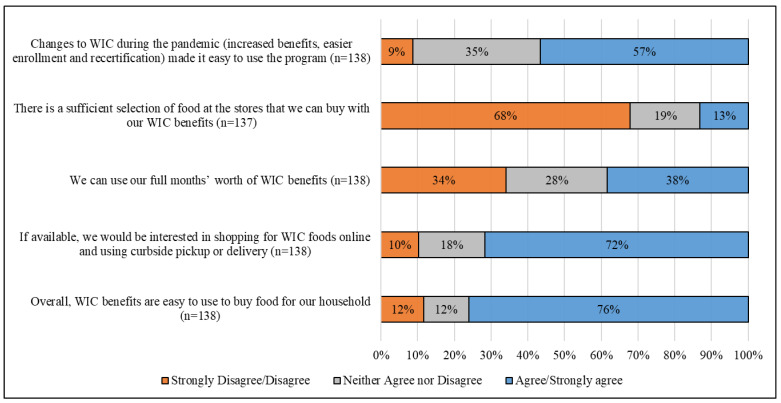
Perceptions of the Special Supplemental Nutrition Program for Women, Infants, and Children (WIC) use during the Coronavirus Disease 2019 (COVID-19) pandemic ^†^. ^†^ Respondent numbers across questions vary due to non-response.

In addition to open-ended questions, respondents shared their views about participating in SNAP or WIC during the pandemic by answering a series of Likert-scale items. These responses are summarized in Figure 1 and Figure 2. Among respondents participating in SNAP, a majority (71.7%) agreed or strongly agreed with the statement that changes to SNAP during the pandemic made it easy to use the program. A little more than half (54.2%) perceived SNAP benefits as being enough to meet their household’s needs. Most SNAP participants (75.7%) reported being able to use their full months’ worth of benefits, and a little over one-third (38.5%) reported that they could use SNAP benefits to pay for groceries ordered online. Overall, 91.4% of SNAP participants felt that SNAP benefits were easy to use to buy food for their household.

A little more than half (56.5%) of WIC participants agreed or strongly agreed with the statement that changes to WIC during the pandemic made it easy to use the program. Few (13.1%) WIC participants reported that there was a sufficient selection of foods at the store that they could buy with their WIC benefits and, correspondingly, less than half (38.4%) of WIC participants reported that they could use their full months’ worth of WIC benefits. A majority (71.7%) of respondents participating in WIC would be interested in shopping for WIC foods online and using curbside pickup or delivery. Overall, 76.1% of WIC participants felt that WIC benefits were easy to use to buy food tor their household.

## 4. Discussion

A national sample of adults surveyed approximately one year after the onset of the COVID-19 pandemic shared reasons for discontinuing participating in SNAP or WIC nutrition assistance programs through open-ended questions on an online survey platform. Respondents who participated in SNAP or WIC during the COVID-19 pandemic also shared perceptions regarding program participation and barriers encountered, such as challenging shopping experiences, through open-ended and closed-ended survey questions. Some issues brought up by respondents were unique to the COVID-19 pandemic (i.e., virus exposure concerns and difficulty finding stocked eligible items in store). Other emergent themes echoed perceptions shared by participants pre-pandemic (i.e., the perception that the WIC program is not worth the effort, transportation challenges), though these barriers may have been heightened during the pandemic. Shared themes concerning reasons given for discontinuing participation and experiences participating in SNAP and WIC a year into the pandemic indicate that policy and programmatic adjustments, including extended program flexibilities, increased benefit value, program outreach, re-examination of qualifying thresholds, and expanded online shopping options, may improve participant utilization of both programs.

Perceived barriers expressed by former SNAP and WIC participants, including virus exposure concerns, recertification challenges, and programmatic burdens associated with participating in WIC, demonstrate the importance of physical presence flexibilities and extended certification periods. In response the COVID-19 pandemic, The Families First Coronavirus Response Act (FFCRA) authorized state WIC agencies to waive physical presence requirements for individuals seeking to enroll or re-enroll, defer required in-person assessments (i.e., height/length, weight, and bloodwork), and to issue benefits remotely [9,10]. Similarly, the FFCRA allowed states to waive face-to-face interview requirements for SNAP application or recertification [11]. Raised concerns by survey respondents suggest that the continuation of physical presence flexibilities can improve program accessibility as the risk of community transmission of COVID-19 continues after the pandemic. Such flexibilities that reduce virus exposure for SNAP- and WIC-eligible individuals are particularly important given the disparities in COVID-19 infection rates driven by unequal heightened infection risks occurring among non-White and low-income households [12,13,14,15] who are also disproportionately impacted by food insecurity [16,17]. The literature shows that WIC participants are interested in telehealth options to address documented participant burdens, including travel to WIC clinic locations [18], clinic wait times [19], and finding time for appointments during clinic hours [20,21,22]. As further evidence of the importance of physical presence flexibilities for WIC participants, all states that did not have WIC electronic benefits transfer systems [23] in place prior to the onset of the pandemic and experienced resultant delays in transitioning to remote benefit issuance experienced declines in WIC participation early in the COVID-19 pandemic. [5,24]. Because our sample comprised respondents residing within 47 different states and sample numbers within states were correspondingly fairly small, we were not able to compare responses by respondent state of residence. Extended certification periods may also ease recertification challenges for participants. Most states requested and received USDA waivers to extend SNAP certification periods during the COVID-19 pandemic [25], and some states received waivers to extend WIC certification periods for child beneficiaries [26]; however, many have since expired.

Concerns raised by respondents related to virus exposure, recertification, and participation burdens additionally suggest a need for communication strategies to increase participant awareness of program flexibilities, both in the wake of COVID-19 and for any future program adjustments. It is possible that some respondents who expressed transportation and virus exposure fears as barriers to participation were unaware of remote recertification and benefit issuance options during the pandemic. Other research conducted during the COVID-19 pandemic has demonstrated varying levels of awareness of temporary pandemic-related waivers among WIC and SNAP participants [27,28]. Communication via channels such as state agency websites, social media, and automated texting and phone calls can increase awareness of program changes among participants and eligible individuals, potentially resulting in increased program uptake [29]. In addition, investments in improving the user experience of online platforms and providing technical assistance to participants will help to ensure remote service offerings promote equitable access to program benefits [30].

Additionally, increasing benefit amounts for both SNAP and WIC may improve perceived program usefulness, as indicated by the expressed inadequacy of benefits, the helpfulness of temporary SNAP emergency allotments issued in 2021 [8], and the finding that nearly half of SNAP recipients felt that benefits were not enough to meet their household’s needs. Historical evidence gathered after a 14% increase in SNAP benefit amounts during the Great Recession (2009 to 2013) demonstrated that benefit increases were associated with reduced food insecurity [31], positive impacts on the economy, and reduced poverty among beneficiaries [32,33]. As an example, the extension of temporary increases in WIC Fruit and Vegetable Cash Value Benefit (CVB) [34] amounts for all beneficiary categories (not yet put into place at the time of survey completion) may improve participant perceptions of adequacy of WIC benefits and, in turn, improve retention rates. These CVB increases may be especially impactful in improving retention when infants turn 1, a historically common drop-off point since the WIC food package for children has a lower monetary value than the average food package for infants, particularly those utilizing benefits for purchasing formula [35,36]. Previous research conducted prior to the pandemic indicates that improved benefits amounts would improve participant perceptions of these food assistance programs [36,37,38].

Outreach efforts may also increase participation rates in both SNAP and WIC. Many respondents who discontinued participating in either program reported that they wanted to save benefits for others they perceived needed the support more. Targeted outreach, communication, and education campaigns making clear that benefits are available for all who need them may amend such perceptions [29]. We were not able to discern from survey responses whether those individuals who reported discontinuing participating to save benefits for others still qualified for benefits. However, the recurrent pattern that respondents felt that others’ food needs were more important than theirs suggests a need for corresponding communication and education campaigns.

An emergent theme specific to discontinuing participation in SNAP was a failure to requalify. A number of respondents who discontinued participation in SNAP shared that they were unsure why they were denied during recertification, expressing a need for the continued nutrition supports. In a qualitative study conducted in Minnesota during the COVID-19 pandemic, several respondents who reported being food-insecure also shared that they failed to qualify for SNAP benefits to support their food needs, despite believing they should be eligible [39]. A re-examination of qualifying thresholds as well as how thresholds are calculated can expand the reach of SNAP. For example, the first round of federal pandemic unemployment benefits (USD 600 weekly) authorized under the Coronavirus Aid, Relief, and Economic Security (CARES) Act [40] was counted as unearned income when determining SNAP eligibility [41]. For some households, the receipt of unemployment insurance put them over the income threshold and made them ineligible to continue receiving benefits.

Finally, convergent qualitative and quantitative findings showed a desire for enhanced or available online benefit redemption options. The rapid expansion of the Online Purchasing Pilot (OPP), a nationwide pilot authorizing online purchasing of foods using SNAP benefits, initially launched in 2019, to increase its reach from 6 to 47 states following the onset of the COVID-19 pandemic allowed unprecedented access to online purchasing for SNAP beneficiaries [42]. However, this expansion did not reach all states at the same time (online purchasing was not made available in Louisiana and Montana until spring 2022 and is not available in Alaska as of June 2022), a limited number of retailers participate, nearly all retailers participating in the OPP charge additional fees for online purchases and delivery [43], and access varies by region—with decreased access for participants living in rural areas [44]. A quarter of respondents in our sample, comprising individuals from 47 states and varied regions (19% within rural areas), indicated that they could not use SNAP benefits to purchase groceries online. Stated barriers indicate that continued expansion and improvement of online purchasing options for SNAP beneficiaries can improve access to nutritional supports for eligible households. 

In contrast, as of June 2022, online purchasing and delivery of foods are not broadly available for WIC participants, although ongoing online purchasing and delivery pilots are testing such policies. Many WIC participants within our sample shared that they had trouble finding WIC-eligible items in stores and expressed support for online redemption options; nearly three-quarters of respondents indicated that they would be interested in online ordering. Beneficiaries receiving WIC who participated in other qualitative research during the pandemic similarly shared that limited food availability in stores acted as a barrier to redeeming benefits during the pandemic and shared that they would like online purchasing options [27,39]. In 2020, the United States Department of Agriculture’s (USDA) Food and Nutrition Service (FNS) awarded USD 2.5 million to the Gretchen Swanson Center for Nutrition to develop recommendations for online ordering using WIC benefits, and to award subgrants to WIC state agencies for innovative projects to test online ordering and transactions [45,46]. Other pilots, such as the “Click and Collect” model in which participants can shop for foods online and then pick up items at the store, have been well-received by participants [47,48].

### Strengths and Limitations

Open-ended responses on surveys allowed respondents to write their responses to questions about their use of, or reasons not to use, SNAP or WIC programs. Because of the format, researchers were unable to ask any further probing questions to better understand responses. Future research examining barriers to the use of SNAP and WIC during the COVID-19 pandemic using a qualitative approach could benefit from more in-depth methodologies to gather individual responses, such as in-depth individual interviews and/or focus groups. Additionally, as is the case with most qualitative analyses, we do not know the frequency with which the emergent themes are held by the population under study; none of the respondents had an opportunity to register endorsement of these volunteered views.

The online survey sampled individuals to reflect the US adult population by race/ethnicity. This analysis includes a subsample of only those respondents who participated in SNAP or WIC approximately one year into the pandemic and/or in the year prior to the onset of the pandemic. So, while the full sample was representative of US demographics by race, the subsample included in this analysis was not. Within our sample, 18% of WIC participants identified as Hispanic, while 41% of WIC participants in 2020 identified as Hispanic [49]. Additionally, 15% of respondents participating in SNAP within our sample identified as non-Hispanic Black, while 26% of SNAP participants identified as non-Hispanic Black in the most recently available national program characteristics report [48]. Respondents within our sample also had relatively higher levels of education compared to national program characteristics reports [49,50]. These differences may be related to the survey being only available via an online platform. While the race/ethnicity distribution of our sample was different from that of national participant characteristics, similar barriers and perceptions related to participating in SNAP and WIC have been described in other studies [27,28,39]. The survey instrument has been used extensively in examining WIC and SNAP participation during the COVID-19 pandemic nationally and in multiple states [7,50,51,52,53]. Comparable studies among nationally representative WIC and SNAP populations are needed to confirm our findings. 

Additional limitations due to the online survey platform include underrepresentation of respondents unable to complete surveys in English or Spanish, respondents without access to internet, and respondents lacking the time to complete a survey. Responses may also be influenced by social desirability, recall bias, and misunderstanding of survey questions. Finally, all perceptions were captured at a moment in time and may have fluctuated throughout the pandemic. Future research that explores perceptions of participants in SNAP and WIC programs in later months during the COVID-19 pandemic will provide important new information.

## 5. Conclusions

The SNAP and WIC programs provide vital nutrition supports for individuals at risk of experiencing food and nutrition insecurity. These needs have been compounded by the COVID-19 pandemic. Encouragingly, the majority of SNAP and WIC participants felt that benefits were easy to use during the pandemic. Barriers faced by eligible participants that prevent individuals from participating in the program and/or from redeeming all benefits suggest a need for programmatic and policy adjustments, such as increased program flexibilities, improved benefit adequacy, and expanded online shopping opportunities to ensure equitable access of foods to at-risk individuals in the wake of the COVID-19 pandemic and beyond. 

## Figures and Tables

**Table 1:** Demographics Characteristics of a National Sample of Households that Discontinued Participating in or Participated in the Supplemental Nutrition Assistance Program (SNAP) or the Special Supplemental Nutrition Program for Women, Infants, and Children (WIC) During the Coronavirus Disease 2019 (COVID-19) Pandemic.

Sample Characteristics	Mean (SD) or %			
	Only participated prior to COVID-19		Participated in last four months	
	SNAP participants (n = 146)	WIC participants (n = 149)	SNAP participants (n = 504)	WIC participants (n = 141)
Age (%)				
18–34	46.6	53.2	43.3	64.1
35–54	42.9	37.6	37.0	31.3
55+	10.5	9.22	19.7	4.58
Race (%)				
Non-Hispanic White	66.4	57.7	61.9	69.6
Hispanic	18.5	24.8	18.0	17.7
Non-Hispanic Black	11.6	14.8	15.4	10.6
Asian	2.05	0.67	1.60	1.42
Native American	1.37	2.01	1.00	0.000
Other/Multiple	0.000	0.000	2.18	0.710
Education (%)				
High school or less	27.2	33.6	37.4	34.9
Some college or more	72.8	66.4	62.6	65.1
Household size	3.48 (1.86)	3.47 (1.44)	2.87 (1.55)	3.66 (1.56)
Income (%)				
<USD 10,000	8.33	13.4	23.2	12.3
USD 10,000–USD 24,999	18.9	22.5	33.8	18.5
USD 25,000 to USD 49,999	34.1	33.1	29.6	33.9
USD 50,000+	38.7	31.0	13.4	35.3
Urbanicity (% urban)	83.8	82.9	81.4	85.6

**Table 2:** Emergent Themes For (A) Reasons for Discontinuing Participation in the Supplemental Nutrition Assistance Program (SNAP) or the Special Supplemental Nutrition Program for Women, Infants, and Children (WIC) and (B) Experience Participating in SNAP or WIC Programs During the Coronavirus Disease 2019 (COVID-19) Pandemic.

Theme	Program	Representative Quotes
Reasons for discontinuing participation in SNAP and WIC programs		
A.1. Virus exposure concerns	Both	“Not COVID safe to go in store.” (*21-year-old female SNAP participant from Michigan)* “Did not want to risk going in a small room with lots of others.” *(31-year-old female WIC participant from Texas)* “Just didn’t want to go outside due to the virus. Never made an appointment to do so.” *(26-year-old female WIC participant from North Carolina)*
A.2. Difficulty with recertification processes Subtheme 1: Time Subtheme 2: Technology	Both	“I felt so depressed and lousy to fill out so much [sic] applications.” (*23-year-old female SNAP participant from New Jersey)* “Once food assistance benefits expire it is difficult to reinitiate the process to receive them.” (*63-year-old female SNAP participant from Michigan)* “I have not had the opportunity to reapply before it got canceled because… I became a caretaker and just had no time.” (*28-year-old female SNAP participant from California)* “I didn’t want to have to requalify and didn’t have the time.” *(32-year-old female WIC participant from Kansas)* “Unable to get signed up online.” (*39-year-old female SNAP participant from Kentucky)* “Because of technology.” (*33-year-old male SNAP participant from California)*
A.3. Saving for others	Both	“…there was food on the table. Save SNAP for the less fortunate families.” (*34-year-old male SNAP participant from Colorado)* “I have support from family and friends so [it is] preferable to let it be for others to benefit from it.” *(35-year-old female WIC participant from New York)* “I did not feel I need it as much as someone else.” (*69-year-old female SNAP participant from Ohio)*
A.4. Fail to requalify	SNAP	“I’ve applied but was denied for some reason. I feel like I should meet the requirements.” (*28-year-old female from Missouri)* “I apparently do not qualify as a single mother of 3.” (*28-year-old female from Florida)*
A.5. Perception that program is not worth the effort	WIC	“My kids no longer need formula, so I dropped it.” (*28-year-old female from Florida)* “…it was just too complicated, but we probably should have kept going with it.” *(31-year-old female from Alabama)* “It wasn’t convenient for me.” *(20-year-old female from New York)*
Experience participating in SNAP and WIC programs		
B.1. Insufficient benefit value	Both	“When we would get back pay for unemployment… they would cut our SNAP benefits because we now had money coming in. But we really didn’t have money because we had to pay all our bills we fell behind on so when they would stop our benefits, we still had a hard time purchasing food.” *(32-year-old female SNAP participant from California)* “The allocated amount we receive makes it very difficult to have it last long enough for each month.” *(36-year-old female SNAP participant from New Jersey)* “There’s just not enough.” *(28-year-old female WIC participant from Tennessee)*
B.2. Transportation barriers	Both	“I don’t have a vehicle right now, so I can only get to a grocery store if I pay for a rideshare service. The rates for that ride are so high that I just can’t afford that. If I were able to pay for groceries online with my EBT card and get grocery delivery, I wouldn’t have to worry about that.” *(31-year-old female SNAP participant from Louisiana)* “Not able to get to store due to losing my job because of COVID-19 so no gas money. Plus newborn baby.” *(27-year-old female WIC participant from California)* “I didn’t have time to go grocery shopping, or sometimes money for gas.” *(18-year-old female WIC participant from Texas)*
B.3. Difficulty making online purchases Subtheme 1: Virus exposure concerns Subtheme 2: Lack of fresh food options	SNAP	“If they do offer payment via EBT I can’t find it. It’s not right out in the open for use.” *(32-year-old female from Michigan)* “It is kind of upsetting that we can’t use them [SNAP benefits] for online services. Because then that means we have to actually go to the grocery store which can increase our likelihood of catching COVID-19.” *(20-year-old female from Idaho)* “Getting food delivered is a lot safer during the pandemic and not being able to use the SNAP benefits online has been very stressful because we cannot afford to pay via cash or credit.” *(40-year-old female from California)* “We cannot order groceries through a delivery service [and] we live almost an hour away from all stores. They won’t deliver here as far as I know. I really wish I had access to fresh fruit and veggies more often.” *(25-year-old female from West Virginia)* “I can use it for non-perishables, but nothing else is available in my area.” *(31-year-old bigender person from Wisconsin)*
B.4. Increased benefits are useful	SNAP	“The $30 increase was much needed. Due to the shortages in the stores, prices have been increasing and many time[s] the items you would get from the pantry you could not get.” *(60-year-old male from Ohio)* “Getting used to the increase to maximum benefit… will be hard to go back… when the pandemic max expires. We used to run out of benefits before 2 weeks. Now we make it to the end of the month.” *(45-year-old male from Ohio)*
B.5. Difficulty finding eligible items in store	WIC	“Most of the stores that we visit do not have all the products that we need, and with a small child, it is difficult to visit multiple stores to get what we need.” *(28-year-old female from Tennessee)* “We have about three stores in town that sell WIC approved items, and since the pandemic happened, those items become scarce.” *(30-year-old female from Arizona)*

## Data Availability

The survey used in this study is publicly available and can be accessed at: https://dataverse.harvard.edu/dataverse/foodaccessandcoronavirus (accessed on 28 September 2022).

## References

[1] Wolfson J.A., Leung C.W. (2020). Food insecurity and COVID-19: Disparities in early effects for US adults. Nutrients.

[2] Fitzpatrick K.M., Harris C., Drawve G., Willis D.E. (2021). Assessing food insecurity among US adults during the COVID-19 pandemic. J. Hunger Environ. Nutr..

[3] Niles M.T., Bertmann F., Belarmino E.H., Wentworth T., Biehl E., Neff R. (2020). The early food insecurity impacts of COVID-19. Nutrients.

[4] US Department of Agriculture, Food and Nutrition Services SNAP Data Tables. https://www.fns.usda.gov/pd/supplemental-nutrition-assistance-program-snap.

[5] US Department of Agriculture, Food and Nutrition Services WIC Data Tables. https://www.fns.usda.gov/pd/wic-program.

[6] Niles M., Neff R., Biehl E., Bertmann F., Belarmino E.H., Acciai F., Ohri-Vachaspati P. Food Access and Food Security during COVID-19 Survey- Version 2.1; Harvard Dataverse, V3. https://dataverse.harvard.edu/dataset.xhtml?persistentId=doi:10.7910/DVN/4KY9XZ.

[7] Niles M.T., Beavers A.W., Clay L.A., Dougan M.M., Pignotti G.A., Rogurs S., Savoie-Roskos M.R., Schattman R.E., Zack R.M., Acciai F. (2021). A Multi-Site Analysis of the Prevalence of Food Insecurity in the United States, before and during the COVID-19 Pandemic. Curr. Dev. Nutr..

[8] US Department of Agriculture, Food and Nutrition Services SNAP Benefits—COVID-19 Pandemic and Beyond. https://www.fns.usda.gov/snap/benefit-changes-2021.

[9] US Department of Agriculture, Food and Nutrition Services WIC—Physical Presence Waiver. https://www.fns.usda.gov/disaster/pandemic/covid-19/wic-physical-presence-waiver.

[10] US Department of Agriculture, Food and Nutrition Services WIC—Remote Benefit Issuance Waiver. https://www.fns.usda.gov/disaster/pandemic/covid-19/wic-remote-benefit-issuance-waiver.

[11] US Department of Agriculture, Food and Nutrition Services PL 116-159—Continuing Resolution SNAP State Options. https://www.fns.usda.gov/snap/cr-state-options.

[12] Samuel L.J., Gaskin D.J., Trujillo A., Szanton S.L., Samuel A., Slade E. (2021). Race, ethnicity, poverty and the social determinants of the coronavirus divide: US county-level disparities and risk factors. BMC Public Health.

[13] Zelner J., Trangucci R., Naraharisetti R., Cao A., Malosh R., Broen K., Masters N., Delamater P. (2021). Racial disparities in coronavirus disease 2019 (COVID-19) mortality are driven by unequal infection risks. Clin. Infect. Dis..

[14] Raifman M.A., Raifman J.R. (2020). Disparities in the population at risk of severe illness from COVID-19 by race/ethnicity and income. Am. J. Prev Med..

[15] Rozenfeld Y., Beam J., Maier H., Haggerson W., Boudreu K., Carlson J., Medows R. (2020). A model of disparities: Risk factors associated with COVID-19 infection. Int. J. Equity Health..

[16] Morales D.X., Morales S.A., Beltran T.F. (2021). Racial/ethnic disparities in household food insecurity during the COVID-19 pandemic: A nationally representative study. J. Racial Ethn. Health Disparit..

[17] Lauren B.N., Silver E.R., Faye A.S., Rogers A.M., Woo-Baidel J.A., Ozzanne A.M., Hur C. (2021). Predictors of households at risk for food insecurity in the United States during the COVID-19 pandemic. Public Health Nutr..

[18] Rossin-Slater M. (2013). WIC in your neighborhood: New evidence on the impacts of geographic access to clinics. J. Public Econ..

[19] Vehawn J., Richards R., West J.H., Cougar Hall P., Crookston B.T., Neiger B.L. (2014). Identifying barriers preventing Latina women from accessing WIC online health information. J. Immigr Minor. Health.

[20] Almeida R., Gutierrez S.A., Whaley S.E., Ventura A.K. (2020). A qualitative study of breastfeeding and formula-feeding mothers’ perceptions of and experiences in WIC. J. Nutr. Educ. Behav..

[21] Bleich S., Dunn C., Kenney E., Fleischhacker S. Strengthening WIC’s Impact during and after the COVID-19 Pandemic. Healthy Eating Research. https://healthyeatingresearch.org/research/strengthening-wics-impact-during-and-after-the-covid-19-pandemic.

[22] Barnes C., Petry S. (2021). “It Was Actually Pretty Easy”: COVID-19 Compliance Cost Reductions in the WIC Program. Public Adm. Rev..

[23] US Department of Agriculture, Food and Nutrition Services WIC—Electronic Benefits Transfer (EBT) and Management Information Systems (MIS). https://www.fns.usda.gov/wic/wic-electronic-benefits-transfer-ebt#:~:text=WIC%20EBT%20is%20an%20electronic,for%20state%20agency%20partnership%20activities.

[24] Vasan A., Kenyon C.C., Roberto C.A., Fiks A.G., Venkataramani A.S. (2021). Association of Remote vs In-Person Benefit Delivery with WIC Participation during the COVID-19 Pandemic. JAMA.

[25] US Department of Agriculture, Food and Nutrition Services SNAP—Extending Certification Periods and Adjusting Periodic Reports. https://www.fns.usda.gov/snap/extending-certification-periods-and-adjusting-periodic-reports.

[26] US Department of Agriculture, Food and Nutrition Services WIC—Extended Certifications. https://www.fns.usda.gov/wic/extended-certifications.

[27] McElrone M., Zimmer M.C., Steeves E.T.A. (2021). A Qualitative Exploration of Predominantly White Non-Hispanic Tennessee WIC Participants’ Food Retail and WIC Clinic Experiences during COVID-19. J. Acad. Nutr. Diet..

[28] Barnes C., Duke Sanford Center for Child & Family Policy (2021). Improving Access to Critical Nutrition Assistance Programs. https://childandfamilypolicy.duke.edu/wp-content/uploads/sites/2/2021/07/Improving-Access-Nutrition-Assistance-brief_5.2021_CBarnes.pdf.

[29] US Department of Agriculture, Food and Nutrition Services SNAP Outreach. https://www.fns.usda.gov/snap/outreach.

[30] Arm K., Miller L. Federal Nutrition Programs during the COVID-19 Pandemic: SNAP. Healthy Eating Research. https://healthyeatingresearch.org/research/federal-nutrition-programs-during-the-covid-19-pandemic-snap/.

[31] Nord M., Prell M. (2011). Food Security Improved Following the 2009 ARRA Increase in SNAP Benefits.US Department of Agriculture, Economic Research Service. https://www.ers.usda.gov/publications/pub-details/?pubid=44839.

[32] Bleich S., Dunn C., Fleischhacker S. (2020). The Impact of Increasing SNAP Benefits on Stabilizing the Economy, Reducing Poverty and Food Insecurity Amid COVID-19 Pandemic. Healthy Eating Research. https://healthyeatingresearch.org/research/the-impact-of-increasing-snap-benefits-on-stabilizing-the-economy-reducing-poverty-and-food-insecurity-amid-covid-19-pandemic.

[33] Fleischhacker S., Bleich S.N. (2021). Addressing Food Insecurity in the United States during and after the COVID-19 Pandemic: The Role of the Federal Nutrition Safety Net. J. Food Law Policy.

[34] US Department of Agriculture, Food and Nutrition Services Implementation of the Extending Government Funding and Delivering Emergency Assistance Act Temporary Increase in the CVV Benefit for Fruit and Vegetable Purchases. https://www.fns.usda.gov/wic/extending-government-funding-and-delivering-emergency-assistance.

[35] Whaley S.E., Martinez C.E., Paolicelli C., Ritchie L.D., Weinfield N.S. (2020). Predictors of WIC participation through 2 years of age. J. Nutr. Educ. Behav..

[36] Weber S., Uesugi K., Greene H., Bess S., Reese L., Odoms-Young A. (2018). Preferences and perceived value of WIC foods among WIC caregivers. J. Nutr. Educ. Behav..

[37] Blumenthal S.J., Hoffnagle E.E., Leung C.W., Lofink H., Jensen H.H., Foerster S.B., Cheung L.W., Nestle M., Willett W.C. (2014). Strategies to improve the dietary quality of Supplemental Nutrition Assistance Program (SNAP) beneficiaries: An assessment of stakeholder opinions. Public Health Nutr..

[38] Haynes-Maslow L., Hardison-Moody A., Patton-Lopez M., Prewitt T.E., Byker Shanks C., Andress L., Osborne I., Jilcott Pitts S. (2020). Examining rural food-insecure families’ perceptions of the Supplemental Nutrition Assistance Program: A qualitative study. Int. J. Environ. Res. Public Health.

[39] Larson N., Alexander T., Slaughter-Acey J.C., Berge J., Widome R., Neumark-Sztainer D. (2021). Barriers to Accessing Healthy Food and Food Assistance during the COVID-19 Pandemic and Racial Justice Uprisings: A Mixed-Methods Investigation of Emerging Adults’ Experiences. J. Acad. Nutr. Diet..

[40] (2020). Coronavirus Aid, Relief, and Economic Security Act, S 3548, 116th Congress, 2nd Session. https://www.congress.gov/bill/116th-congress/senate-bill/3548/text.

[41] Center on Budget and Policy Priorities Pandemic Unemployment Insurance Provisions: What They Mean for Access to SNAP, Medicaid, and TANF. https://www.cbpp.org/research/economy/pandemic-unemployment-insurance-provisions-what-they-mean-for-access-to-snap.

[42] US Department of Agriculture, Food and Nutrition Services Stores Accepting SNAP Online. https://www.fns.usda.gov/snap/online-purchasing-pilot.

[43] Headrick G., Khandpur N., Perez C., Smith Taillie L., Bleich S.N., Rimm E.B., Moran A. (2021). Content Analysis of Online Grocery Retail Policies and Practices Affecting Healthy Food Access. J. Nutr. Educ. Behav..

[44] Moran A., Headrick G., Khandpur N. (2021). Promoting Equitable Expansion of the SNAP Online Purchasing Pilot. Healthy Eating Research. https://healthyeatingresearch.org/research/promoting-equitable-expansion-of-the-snap-online-purchasing-pilot/.

[45] US Department of Agriculture, Food and Nutrition Services WIC Supports Online Ordering and Transactions in WIC. https://www.fns.usda.gov/wic/supports-online-ordering-transactions.

[46] Gretchen Swanson Center for Nutrition WIC Online Ordering Grant. https://www.centerfornutrition.org/wic-online-ordering.

[47] Zimmer M.C., Beaird J., Steeves E.T.A. (2021). WIC participants’ perspectives about online ordering and technology in the WIC program. J. Nutr. Educ. Behav..

[48] Zimmer M., McElrone M., Steeves E.T.A. (2021). Feasibility and Acceptability of a “Click & Collect” WIC Online Ordering Pilot. J. Acad. Nutr. Diet..

[49] US Department of Agriculture, Food and Nutrition Services WIC Participant and Program Characteristics 2020. https://www.fns.usda.gov/wic/participant-program-characteristics-2020.

[50] US Department of Agriculture, Food and Nutrition Services Characteristics of SNAP Households: FY 2019. https://www.fns.usda.gov/snap/characteristics-snap-households-fy-2019.

[51] Ohri-Vachaspati P., Acciai F., De Weese R.S. (2021). SNAP participation among low-income US households stays stagnant while food insecurity escalates in the months following the COVID-19 pandemic. Prev. Med. Rep..

[52] Harper K., Belarmino E.H., Acciai F., Bertmann F., Ohri-Vachaspati P. (2022). Patterns of Food Assistance Program Participation, Food Insecurity, and Pantry Use among US Households with Children during the COVID-19 Pandemic. Nutrients.

[53] Clay L.A., Rogus S. (2021). Food access worries, food assistance use, purchasing behavior, and food insecurity among New Yorkers during COVID-19. Front. Nutr..

